# Aberrant Resting-State Corticostriatal Functional Connectivity in Cirrhotic Patients with Hyperintense Globus Pallidus on T1-Weighted MR Imaging

**DOI:** 10.1371/journal.pone.0048886

**Published:** 2012-11-07

**Authors:** Xi-Qi Zhu, Hua-Jun Chen, Yu Wang, Ying Cui, Gao-Jun Teng

**Affiliations:** 1 Jiangsu Key Laboratory of Molecular and Functional Imaging, Department of Radiology, Zhongda Hospital, Medical School, Southeast University, Nanjing, China; 2 Department of Radiology, The Second Hospital of Nanjing, Medical School, Southeast University, Nanjing, China; University of Maryland, United States of America

## Abstract

Neurobiological and neuroimaging studies have emphasized the structural and functional alterations in the striatum of cirrhotic patients, but alterations in the functional connections between the striatum and other brain regions have not yet been explored. Of note, manganese accumulation in the nervous system, frequently reflected by hyperintensity at the bilateral globus pallidus (GP) on T1-weighted imaging, has been considered a factor affecting the striatal and cortical functions in hepatic decompensation. We employed resting-state functional magnetic resonance imaging to analyze the temporal correlation between the striatum and the remaining brain regions using seed-based correlation analyses. The two-sample t-test was conducted to detect the differences in corticostriatal connectivity between 44 cirrhotic patients with hyperintensity at the bilateral GP and 20 healthy controls. Decreased connectivity of the caudate was detected in the anterior/middle cingulate gyrus, and increased connectivity of the caudate was found in the left motor cortex. A reduction in functional connectivity was found between the putamen and several regions, including the anterior cingulate gyrus, right insular lobe, inferior frontal gyrus, left parahippocampal gyrus, and anterior lobe of the right cerebellum; increased connectivity was detected between the putamen and right middle temporal gyrus. There were significant correlations between the corticostriatal connectivity and neuropsychological performances in the patient group, but not between the striatal connectivity and GP signal intensity. These alterations in the corticostriatal functional connectivity suggested the abnormalities in the intrinsic brain functional organiztion among the cirrhotic patients with manganese deposition, and may be associated with development of metabolic encephalopathy. The manganese deposition in nervous system, however, can not be an independent factor predicting the resting-state brain dysfunction in real time.

## Introduction

Metabolic encephalopathy caused by liver cirrhosis includes a wide spectrum of neurological dysfunction, such as progressive deficits in neuromotor function, cognition, and intellect. Subcortical structures, the basal ganglia in particular, are implicated in brain dysfunction induced by hepatic dysmetabolism. Previous studies have demonstrated that lesions of the striatum are involved in chronic acquired hepatocerebral degeneration [1], [2], [3]. For example, Finlayson and Supeville [4] investigated the neuronal cell loss in basal ganglia samples obtained at autopsy from cirrhotic patients. Guevara and colleagues [5] showed a remarkable decrease in the gray matter density of the putamen coupled with atrophy of the cerebral cortex. Additionally, an increased expression of peripheral benzodiazepine binding sites was observed in the striatum and prefrontal regions of cirrhotic patients with minimal hepatic encephalopathy (MHE) [6].

Of note, many patients with cirrhosis have bilateral symmetric hyperintensity of the globus pallidus on T1-weighted MR imaging, reflecting the presence of manganese deposition in the nervous system [7], [8], [9], [10]. It has been shown that manganese accumulation in the brain can directly induce disturbances in the astrocytes and neuron function [11], [12]. In addition, Butterworth and colleagues [13] found that the toxic effects of manganese may lead to alterations in the dopaminergic neurotransmitter system in the striatum. It is suggested that the selective loss of the dopamine D_2_ receptor in the globus pallidus and putamen plays a key role in the development of hepatic encephalopathy (HE) [14], [15]. Furthermore, recent data indicate that manganese deposition in brain is related to the presence of parkinsonism symptoms in cirrhotic patients [16]. Therefore, manganese accumulation in brain tissues has been considered as a factor affecting the striatal and cortical functions in hepatic decompensation and involves in the mechanism of metabolic encephalopathy in cirrhotic patients [9], [10], [17].

It is well known that there are anatomical and functional connections between the cortex and striatal structures: the cerebral cortex projects to the basal ganglia and, from there, to the thalamus and back to the cortex, forming a corticostriatal network. A recent study using diffusion tensor imaging has further demonstrated the segregation of corticostriatal connections in vivo, taking into consideration the different compartments of the striatum [18]. Many studies have pointed to the functional distinctions between the classical basal ganglia structures and the independent corticostriatal circuits, such as the motor, executive, visual and motivational loops; these findings have been confirmed [19]. For example, the motor loop connects the putamen to the cerebral motor cortex, including the primary motor cortex, primary somatosensory cortex, and premotor and supplementary motor cortices; the executive loop consists of the anterior dorsal striatum and interacting regions of the dorsal and lateral portions of the prefrontal cortex. Therefore, impairments in the striatum can always induce aberrant connections with the cerebral cortex, resulting in various neuropsychological diseases, such as attention deficit/hyperactivity disorder [20], [21], Parkinson’s disease [22], [23], autism [24] and obsessive-compulsive disorder [25].

Resting-state functional connectivity, defined as the temporal correlations between neuronal activities in remote brain regions, has been increasingly used to investigate the connectivity strength of brain networks by measuring coherent, spontaneous fluctuations in the blood oxygenation level dependent signal [26], [27], [28]. It is additionally suggested that the temporal coherence between different regions can also reflect brain anatomical connectivity [29]. Thus, large-scale functional connectivity, at least in part, represents the model of the brain structural and functional organization in human beings. Notably, by the resting-state functional connectivity technique, Di Martino and colleagues [30] have reproduced the multiple patterns of corticostriatal circuits that are anatomically connected, which provides compelling evidence about the functional organization of the basal ganglia. Importantly, disruption of the resting-state functional connection within the corticostriatal loops can predict the presence of neurological diseases, such as obsessive-compulsive disorder [25] and Parkinson’s disease [22]. However, there has not yet been a report about the alterations in the resting-state functional connectivity of the striatum among the patients with cirrhosis.

In the present study, we aimed to test whether the temporal correlation of neuronal activity between the striatum and other brain regions changes in the cirrhotic patients with manganese accumulation in their nervous systems, and whether the altered functional connectivity of the striatum correlates with neurocognitive dysfunction in these patients.

## Patients and Methods

### Participants

This study is approved by the institutional Ethics Committee of Southeast University, Nanjing, China. A total of 64 right-handed subjects were included in this study; they gave written informed consent before participation. The 44 cirrhotic patients were matched with the 20 healthy controls with respect to age, sex, and education year. The diagnosis of cirrhosis was based on a liver biopsy and/or clinical criteria that include physical, laboratory, and imaging examinations. Two experienced neuroradiologists identified the patients with hyperintensity in the bilateral GP. The clinical characteristics of all subjects are summarized in Table 1.

**Table 1:** The clinical characteristics of all subjects.

Clinical variables	Healthy Controls(*n* = 20)	Patients with Cirrhosis(*n* = 44)	*p* value
Age (years)	51.5±7.7	50.4±10.1	0.675
Education (years)	8.3±2.5	7.9±3.0	0.593
Sex (male/female)	16/4	39/5	0.594 (χ^2^-test)
Child–Pugh stage (A/B/C)	–	8/20/16	–
Etiology (HBV/alcoholism/both/other)	–	35/1/4/4	–
Trail making test A (second)	47.4±14.4	59.8±22.6	0.027
Trail making test B (second)	111.2±27.6	146.9±48.4	0.001
Digit symbol test (raw score)	43.3±9.1	32.4±12.0	0.001
Block design test (raw score)	31.2±7.9	24.0±8.5	0.002

No patient currently presented with HE or other neurological or psychiatric disorders, took psychotropic medications, or suffered from uncontrolled endocrine or metabolic diseases (e.g., diabetes mellitus and hyperthyroidism) when they were recruited. The subjects were excluded if they had visual deficits that resulted in a disability to complete the neuropsychological tests and the patients had to be abstinent from alcohol at least 6 months before the study.

### Neuropsychological Tests

A battery of neuropsychological tests was administrated to all subjects to assess each individual’s neuropsychological status. These neuropsychological tests included the trail-making test-A, trail-making test-B, digit symbol test and block design test. The digit symbol test and block design test are subtests in the Chinese Version of the Wechsler Adult Intelligence Scale Revised. These tests have typically been applied to detect mild neuropsychological impairments in cirrhotic patients [17], [31].

### MR Data Acquisition

All MR images were acquired in a 1.5 T system (Vantage Atlas, TOSHIBA), equipped with 30 mT/m gradients and a standard quadrature head coil. All subjects were instructed to lie still with their eyes closed, avoid falling asleep, and think of nothing in particular. A gradient echo planar imaging sequence was used to obtain functional data (TR = 2500 ms, TE = 40 ms, flip angle = 90°, 22 axial slices with 5 mm thickness and 1 mm gap, a total of 120 brain volumes, 64×64 matrix, 3.75×3.75 mm^2^ in-plane resolution). High-resolution anatomical images were collected by a gradient echo pulse sequence in the sagittal plane (TR = 12 ms, TE = 5 ms, TI = 300 ms, flip angle = 20°, 108 slices with 1.5 mm thickness, 1×1 mm^2^ in-plane resolution). Additionally, routine T1- and T2-weighted fast spin-echo images were also acquired to rule out any incidental pathological abnormalities. Using the routine T1-weighted images, two experienced radiologists (Xi-Qi Zhu and Gao-Jun Teng who respectively had 10 and 15 years of imaging experience) identified whether the cirrhotic patients had hyperintensity in the bilateral GP.

### The Calculation of the Bilateral GP Signal Intensity

The calculations of the bilateral GP signal intensity on T1-weighted images were processed using Statistical Parametric Mapping (SPM5, http://www.fil.ion.ucl.ac.uk/spm). High-resolution T1-weighted images were normalized to the standard SPM-T1 template. A mask of the bilateral GP was manually created from the normalized T1-weighted images of the controls, using the MRIcro software (http://www.mccauslandcenter.sc.edu/mricro/mricro). Then, the signal intensity of the bilateral GP from T1-weighted images was calculated for each subject using the created GP-ROI mask. Finally, the independent-sample student t-test was performed to determine the difference in the signal intensity of the GP between the patients and controls, using SPSS v.15.0 (SPSS, Chicago, IL, USA). A *p* value <0.05 was considered statistically significant.

### Functional Data Preprocessing

The functional MRI data were preprocessed using the Data Processing Assistant for Resting-State fMRI (DPARSF) software package [32], SPM5 and Resting-State fMRI Data Analysis Toolkit (REST, http://www.restfmri.net). The first 8 functional volumes of the functional images were discarded for signal equilibrium and subjects’ adaptation to the scanning noise. The data were then corrected for slice timing and head-motion, spatially normalized into the stereotactic space of the Montreal Neurological Institute (MNI), and resampled to a 2 mm isotropic resolution. The participants who had excessive head motion, more than 2.0 mm of maximum displacement in the x, y, or z directions and 2.0 degrees of angular rotation about each axis, were excluded from this study. The data were then spatially smoothed using a Gaussian kernel of 6 mm full width at half-maximum. Finally, linear drift was removed, and a temporal filter (0.01–0.08 Hz) was applied to reduce low-frequency drifts and physiological high-frequency noise.

### Functional Connectivity Analysis

**Figure 1:**
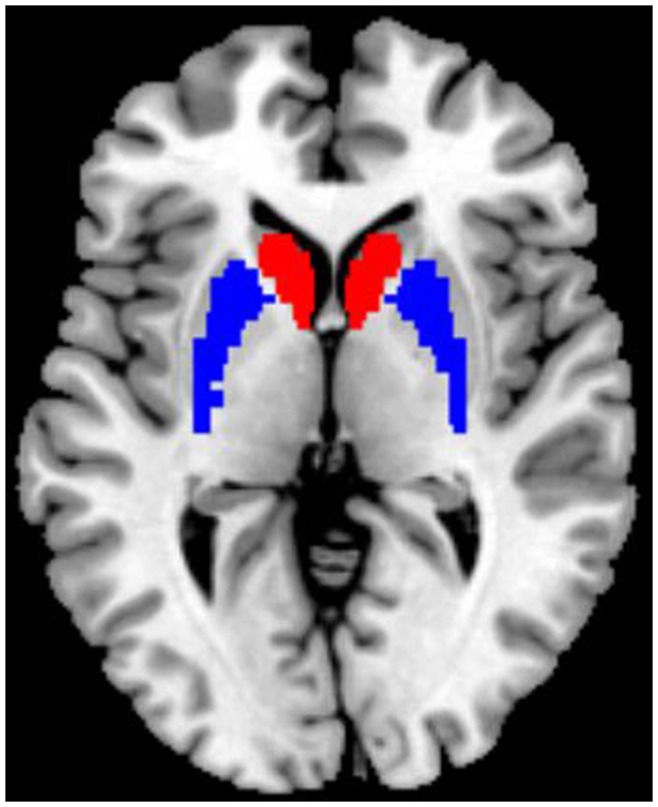
Location of the striatal seed (regions of interest), including the bilateral caudate head (red) and bilateral putamen (blue).

Functional connectivity analyses were performed using REST software. The seed ROIs of the bilateral striatum were generated using the WFU_PickAtlas Tool v.2.4 software (http://www.ansir.wfubmc.edu). Based on previous studies [20], [22], we included two seed ROIs, namely the bilateral caudate head and the bilateral putamen (Figure 1), in this study. The posterior parts of the caudate and GP were not included in our analyses, given the small volume and/or the close proximity to the ventricles that may contaminate the time course with the signal from the cerebrospinal fluid.

The mean time series of each ROI was calculated by averaging the time series of all voxels within the ROI. Then, a voxel-wise correlation analysis was performed between the mean time series of each ROI and the time series of the remaining brain areas. Finally, Fisher’s z-transform was applied to improve the normality of the correlation coefficients. In addition, six head motion parameters and a mean time series of the global, white matter and cerebrospinal fluid signals were included in the regression analysis, to remove the possible effects of such factors on the results.

### Within-group Analyses

Within each group, the individual z-value was entered into a random effect one-sample *t*-test in a voxel-wise manner to determine the regions with significant, positive connectivity to the specific seed. Multiple comparison corrections were performed using the AlphaSim program (written by D. Ward, http://afni.nih.gov/afni/docpdf/AlphaSim.pdf). Additionally, the statistical thresholds were all set at a combined threshold of *p*<0.05 for a single voxel and a minimum cluster size of 531 voxels, yielding a corrected threshold of *p*<0.05, which was determined by Monte Carlo simulations. These analyses were performed only within the gray matter mask.

### Between-group Analyses

For each seed-ROI, a mask was created by combining the two z-maps of both groups, which were the result of one-sample t-tests. Furthermore, the between-group two-sample t-test was constrained within the mask to determine the differences in the functional connectivity distribution of each seed ROI. Age and education year were included as nuisance covariates. A multiple comparisons correction was performed using the AlphaSim program. The thresholds were set at a combined threshold of *p*<0.01 and a minimum cluster size of 100 voxels, yielding a corrected threshold of *p*<0.005.

### Correlation Analysis

To investigate the relationship between the individual z-values and the GP signal intensity in the patient group, Pearson’s correlation analyses were performed in a voxel-wise manner using the REST software. Also, we conducted Pearson’s correlation analyses to determine the regions in which the z-value significantly correlated with the results of neuropsychological tests in the patient group. For all these analyses, the statistical threshold was set at a combined threshold of *p*<0.01 and a minimum cluster size of 100 voxels, yielding a corrected threshold of *p*<0.005. These analyses were performed within the same masks for the between-group analyses.

In addition, Pearson’s correlation analyses, using SPSS v.15.0, were performed to determine the relationships between the bilateral GP signal intensity and the results of neuropsychological tests in the patient group. A *p* value <0.05 was considered statistically significant.

## Results

### Neuropsychological Assessment

The cirrhotic patients presented with significantly worse performance than the healthy controls in all neurocognitive tests (Table 1).

### The Signal Intensity of the Globus Pallidus (GP) on T1-weighted Imaging

**Figure 2:**
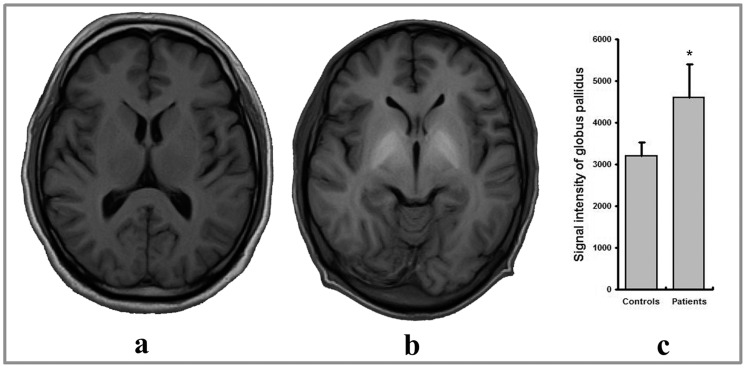
Manganese deposition in a patient with cirrhosis, reflected by the hyperintensity on the transverse T1-weighted image (b), as compared with a healthy control (a). (c) showed that the signal intensity at globus pallidus in the patient group was significantly higher than that of healthy controls. *indicates a *p* value <0.001.

All cirrhotic patients showed remarkable hyperintensity at the bilateral GP on routine T1-weighted imaging. The GP signal intensity in the patient group was significantly higher than that of healthy controls (Figure 2).

### Within-group Analysis of Functional Connectivity

**Figure 3:**
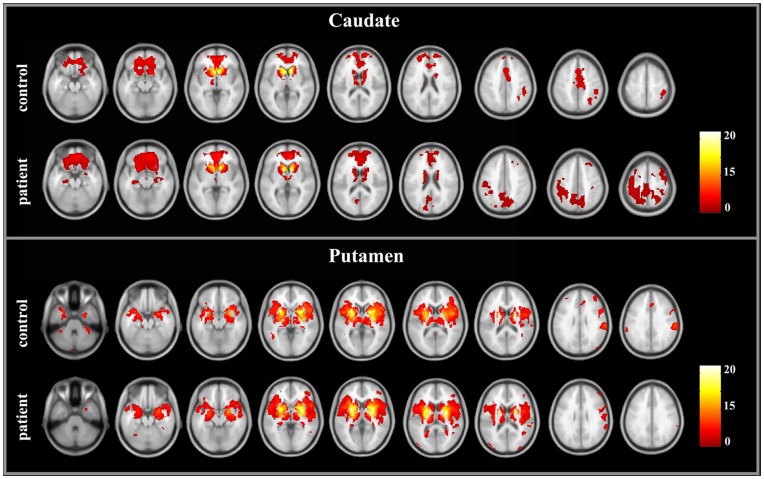
Brain regions showing the significant connectivity to the bilateral caudate and putamen in the cirrhotic patients and healthy controls.

We examined the positive functional connectivity pattern of the caudate. In the control group, the regions showing positive functional connectivity with the bilateral caudate included the bilateral caudate, thalamus, medial frontal gyrus, anterior cingulate gyrus, superior and middle frontal gyri, inferior frontal gyrus, and right inferior parietal lobule. This pattern was consistent with previous reports [22], [30]. The patients had similar connectivity patterns of the caudate (Figure 3). However, in the patient group, some other regions, including the precuneus/posterior cingulate cortex, bilateral hippocampus, bilateral precentral and postcentral gyri, and bilateral superior frontal gyrus, showed significant connectivity with the caudate as well.

We examined the positive functional connectivity pattern of the putamen. In the control group, a set of regions had positive functional connectivity with the bilateral putamen, including the bilateral putamen, caudate, GP, thalamus, insula lobe, medial temporal cortex, anterior cingulate gyrus, inferior frontal gyrus, superior frontal gyrus, precentral gyrus, postcentral gyrus, and anterior cerebellum lobules. This pattern was also consistent with previous reports [20], [30]. There was the similar pattern of the putamen connectivity in the patient group (Figure 3).

### Between-group Analysis of Functional Connectivity

**Figure 4:**
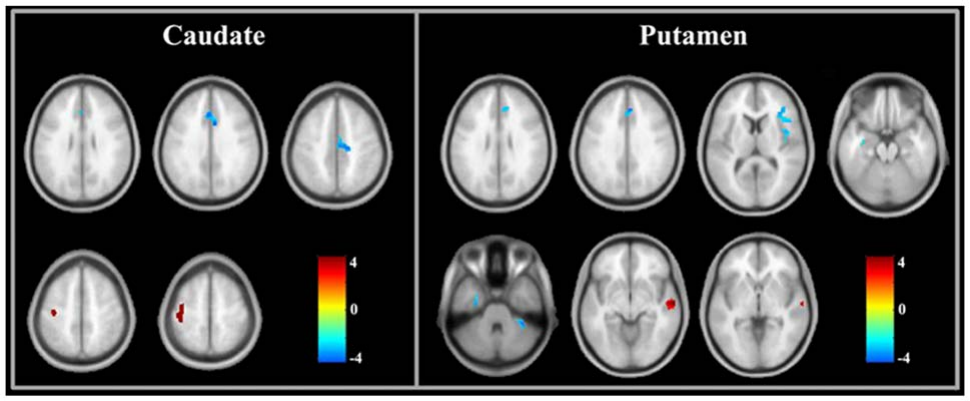
Brain regions showing significant differences in corticostriatal functional connectivity between patients and controls. Red indicates increased connectivity and blue indicates decreased connectivity.

Figure 4 and Table 2 show the results of the between-group analyses on the corticostriatal functional connectivity. Compared to healthy controls, the cirrhotic patients primarily showed decreased corticostriatal functional connectivity, although there was an increase in the functional connectivity between some regions and the striatum. A decreased functional connectivity of the caudate was detected in the anterior/middle cingulate gyrus, and an increased connectivity of the caudate was found in the left motor cortex. The patient group showed a significant decrease in the functional connectivity between the putamen and several regions, including the anterior cingulate gyrus, right insular lobe, right inferior frontal gyri, left parahippocampal gyrus, and right cerebellum anterior lobe. Additionally, there was increased functional connectivity between the putamen and right middle temporal gyrus.

**Table 2:** Regions showing significant differences in the corticostriatal functional connectivity between patients and controls.

Regions	Voxels	BA	MNI coordinates			Peak t-value
			x	y	z	
**1) Seed-ROI: caudate**						
**Decreased functional connectivity**						
B Anterior cingulate gyrus	215	32/24	6	12	34	−3.38
R Middle cingulate gyrus	135	24	4	−14	44	−3.17
**Increased functional connectivity**						
L Precentral and postcentral gyri	214	4/3	−40	−18	56	3.07
**2) Seed-ROI: putamen**						
**Decreased functional connectivity**						
B Anterior cingulate gyrus	103	32	6	34	32	−3.66
L Parahippocampal gyrus	102	20/36	−36	−12	−14	−3.21
R Inferior frontal gyrus	217	45	38	32	8	−3.30
R Insula lobe	132	13/44	46	2	6	−3.27
R Cerebellum anterior lobe	114		34	−40	−34	−3.69
**Increased functional connectivity**						
R Middle temporal gyrus	174	21	54	−12	−12	3.84

Note: L,R, and B: left, right and bilateral, respectively. BA: Brodmann area.

### Results of the Correlation Analyses

**Figure 5:**
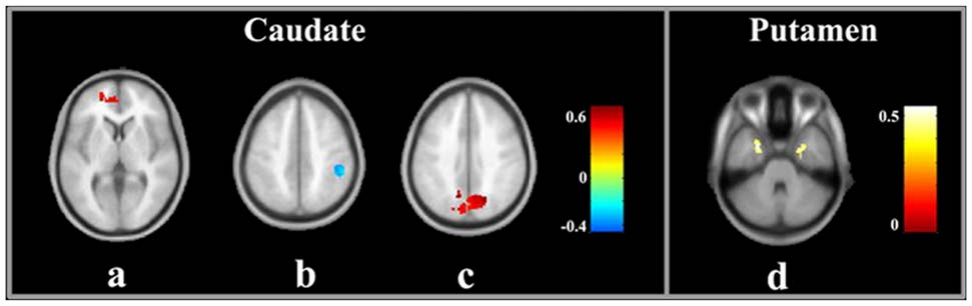
The regions in which the strength of corticostriatal functional connectivity significantly correlate with the neurological performance in trail-making test A (a), trail-making test B (b, d), and block design test (c). Red indicates positive correlation and blue indicates negative correlation.

There was no correlation between the strength of the striatal connectivity and the GP signal intensity in the patient group. Furthermore, we did not find a correlation between the bilateral GP signal intensity and the results of the neuropsychological tests. However, the connectivity strengths between the striatum and several brain regions (Figure 5) were correlated with the neuropsychological performances of the cirrhotic patients.

## Discussion

In the present study, we detected alterations in the resting-state functional connectivity within the caudate-cortical and putamen-cortical circuits in the cirrhotic patients with hyperintense globus pallidus on T1-weighted MR imaging that reflected manganese accumulation in nervous systems [7], [8], [9], [10]. These abnormalities may affect the brain’s functional integration and partially account for the cirrhotic patients’ deficits in cognition. To the best of our knowledge, this is the first study exploring the corticostriatal functional connectivity in cirrhotic patients with manganese deposition.

Neurobiological and neuroimaging studies have revealed the structural and functional alterations in the striatum of cirrhotic patients [3], [4], [12], [14]. Of note, manganese accumulation in the nervous system could exert a damaging effect on the pathological function of the striatum in patients with cirrhosis [14], [15]. Furthermore, it was written in a review that chronic exposure to manganese can result in selective dopaminergic dysfunction, neuronal loss, and gliosis in the basal ganglia structures together with characteristic astrocytic changes known as Alzheimer type II astrocytosis [12]. The influences of manganese neurotoxicity have also been described in people with occupational exposure to manganese that had GP hyperintensity as well [33]. However, it was noted that the striatal damages due to hepatic decompensation could couple with the alterations within other brain regions. For example, the PET study revealed the relative increment of cerebral blood flow in the striatum and cerebellum, concomitant with decreased cerebral blood flow in the cerebral cortex [34], [35]. In addition, a similarly altered pattern of cerebral glucose metabolism in patients with severe liver disease has been reported [34], [36]. This redistribution of the cerebral blood flow and glucose metabolism contributes to metabolic encephalopathy [34], [35]. Taken together, the selective vulnerability of the striatum and compelling abnormalities in the distinct cortical regions probably offer a cogent explanation for the aberrant resting-state corticostriatal functional connectivity in cirrhotic patients with manganese deposition.

However, we did not find a correlation between the GP signal intensity and abnormal corticostriatal functional connectivity or neuropsychiatric performances, although the cirrhotic patients had significantly higher signal intensity at the bilateral GP than healthy controls. This result was consistent with a previous finding demonstrating that there was no significant correlation between the GP hyperintensity and neuropsychiatric impairments [7] or metabolic disorders [37]. This lack of correlation may be due to the indirect measurements, in which the hyperintensity on T1-weighted images represents abnormal manganese deposition [9]. Another explanation for this result is the discrepancy in the time-course across the dynamic changes of neuropsychological function and the GP signal intensity. A longitudinal study showed that patients would still have GP hyperintensity 6 months after successful liver transplantation, despite quick resolution of neurological functions [38]. In addition, this result suggests, at least in part, that manganese deposition may not be an independent factor predicting neurological disorder in cirrhotic patients, although manganese toxicity can induce abnormalities in the striatum and the relevant cortex. Additionally, some other clinical variables, such as the severity of liver disease and the history of an HE episode, may be associated with the aberrant brain functional organization as well because more serious cerebral impairments have been found in the patients with higher Child-Pugh stages or histories of overt HE [5].

In the patient group, decreased functional connectivity was found between the anterior cingulate cortex (ACC) and striatum, including the caudate and putamen. The ACC is an important center subserving the brain’s attention-related network (e.g., executive control network) [39]. There has been evidence indicating the structural, metabolic and functional abnormalities of the ACC in cirrhotic patients [5], [40], [41]. Also, the IFG and anterior insular, two additional important regions engaging human attention [42], [43], showed decreased connectivity to the putamen. The decreased connection between these cortical regions and the striatum may be a possible mechanism underlying attention deficit one of most frequent cognitive impairments in cirrhotic patients [44]. In addition, the reduction in the functional connectivity between the left hippocampus and putamen may be associated with deficits in visual memory as well as executive dysfunction, considering that the score in trail-making test-B was positively correlated with the connectivity strength of the putamen to the bilateral hippocampus (Figure 5).

Alternatively, several regions, including the left precentral/postcentral gyrus and right middle temporal gyrus, had abnormal increments of corticostriatal functional connectivity. The precentral and postcentral gyri are important components of the motor and sensory areas, and the middle temporal gyrus is a relay center processing visual information. Additionally, it was noted that these patients had significant correlations between the caudate and some regions within the default-mode network (DMN) [45], such as the precuneus/posterior cingulate cortex and bilateral hippocampus (Figure 3). The DMN is an intrinsic network showing task-induced deactivation to reallocate brain resources to complete specific behavioral goals. It is associated with various types of cognitive processes, such as attention and memory retrieval [42], [45]. Therefore, the aforementioned changes likely reflect the reorganization of the corticostriatal circuits, which may beneficially limit the neuromotor dysfunction (e.g. bradykinesia) and neurocognitive impairments (e.g., visual memory and visuomotor coordination) that are common and early neurological deficits induced by liver decompensation [44].

Interestingly, we found significant correlations between the connectivity measurement and the patients’ neurological performances in several regions that are mainly located within the DMN. Recent data have shown an aberrant intrinsic functional connectivity within the DMN in the case of HE [46]. Thus, abnormalities in the brain’s intrinsic connectivity may be implicated in the pathological mechanisms underlying neurocognitive impairments due to hepatic decompensation. Furthermore, measuring alterations in the striatal functional connectivity with the DMN may be useful in indicating that there are mild brain dysfunctions in the cirrhotic patients, which is made more compelling with assessments by neurological tests.

Several limitations should be noted in this study. First, we did not measure the level of manganese in the blood, although correlations between the biochemical alterations and blood manganese have been consistently reported in patients with liver dysfunction [47], [48]. To comprehensively understand the influences of manganese dysmetabolism on brain dysfunction, additional correlation analyses between the level of blood manganese and resting-state functional connectivity may be necessary in further studies. Second, the variability in the patients’ clinical characteristics, such as the etiology of cirrhosis and the history of overt HE, may decrease the statistical power in our study. For example, it is reported that the different etiologies of liver cirrhosis are associated with varied degrees of impairment in the nervous system. Alcoholism could exert additional effects on the brain, as compared to hepatitis virus infection [49]. In this study, some cirrhotic patients had an etiology of alcohol consumption, although a majority of the patients had HBV inflection as the etiology for their liver cirrhosis (Table 1). Further studies with stratified analyses based on the different clinical variables may be required to investigate the influences of these clinical factors. Third, we also noted that the current investigation focused on the altered corticostriatal functional connectivity in cirrhotic patients with hyperintensity of bilateral GP, using the healthy subjects as controls. However, to better classify specify of this finding, the future studies, which include the cirrhotic patients without hyperintensity of bilateral GP as controls, is also needed. Another limitation is the slow sampling rate (TR = 2.5 s) used in the functional imaging, due to the limited MR device configuration, which may result in the noise from the respiratory and cardiac cycles aliasing into the resting-state low frequency ranges and possibly becoming a potential confounding factor. To reduce these potential artificial effects, estimations can be made by recording the respiratory and cardiac cycles during the functional imaging. This would be recommended in future studies.

In summary, we detected the changes in the functional connection within the cortical-striatal circuits of cirrhotic patients with hyperintense globus pallidus on T1-weighted MR imaging. These alterations in the corticostriatal functional connectivity may indicate there are abnormalities in the intrinsic brain functional organization and may be associated with the development of metabolic encephalopathy in cirrhotic patients. Although hyperintense globus pallidus on T1-weighted MR imaging reflected the manganese accumulation in the nervous system; however, this abnormality can not be an independent, single factor predicting the resting-state brain dysfunction in real time.
